# Seeking support through solidarity: female leader’s experiences of workplace solidarity in male-dominated professions

**DOI:** 10.3389/fpsyg.2023.1119911

**Published:** 2023-06-29

**Authors:** Daphne Pillay-Naidoo, Corlette Vermeulen

**Affiliations:** Department of Human Resource Management, University of Pretoria, Pretoria, South Africa

**Keywords:** female solidarity, male-dominated professions, solidarity interventions, solidarity barriers, solidarity benefits

## Abstract

**Introduction:**

While there is a plethora of research that documents the numerous barriers affecting female leaders in the modern workplace, there is a lack of literature which focuses on strategies or motivating resources that women use to navigate the workplace environment. Despite facing significant barriers in their leadership journeys, there are female leaders who are able to overcome these barriers to achieve leadership positions. These women leaders draw on personal and external motivating factors to assist them in dealing with the challenges associated with being a female leader as a result, research on motivating strategies for women’s career progression is a research topic that warrants immediate attention. Female solidarity as a motivating resource has been gaining traction in the field of leadership studies and can be seen as a supportive resource that can be used by current and aspiring female leaders to progress in underrepresented environments. Although female solidarity is but only one of the many strategies that can be implemented to motivate women in leadership positions, the increase of female solidarity in the workplace is expected to alleviate the conditions that reinforce essentialist notions of the “queen bee syndrome” in which women are seen as unsupportive of each other.

**Method:**

A qualitative research approach was used for this study, following an interpretive descriptive design. A total of 13 semi-structured interviews were conducted with female leaders in male-dominated professions within South Africa. Data was analysed using thematic content analysis.

**Results:**

Results of the study were analyzed in line with three primary content areas, i.e., barriers to female solidarity in the workplace, benefits of female solidarity in the workplace and workplace interventions to increase solidarity.

**Discussion:**

In the context of the study the predominant barriers to female solidarity within male-dominated workplaces were identified as unfair workplace behaviours, generational beliefs, societal expectations, organisational cultures, stereotypes and stigmas. The benefits of female solidarity within male-dominated workplaces were identified as career shaping mentorship, female recognition, female representation and female support. Lastly, the interventions that can be implemented to increase female solidarity within male-dominated workplaces were conceptualised as networking, transforming the company culture, socialisation and mentorship.

## Introduction

Researchers in female leadership (Hideg and Shen, 2019; Herbst, 2020; Liu et al., 2022; Pillay-Naidoo and Nel, 2022) argue that while the 21st century has seen an influx in the number of women entering the paid labor force, women remain underrepresented in leadership positions. In a report published in 2017, findings indicated that women accounted for only 17% of corporate board members and 12% of executive committee members in the top 50 listed G-20 companies within Europe (Devillard et al., 2018). Grant Thornton International Ltd. (2015) states that only 22% of senior management positions are occupied by women globally. Women constantly face challenges such as senior managerial underrepresentation within the private sector despite accounting for nearly 50% of the workforce on aggregate worldwide (Statistics New Zealand, 2015).

Drawing the concept of underrepresentation of women leaders to the South African context, Statistics South Africa (2017) suggests that despite 52% of the South African working population consisting of women, merely 22% of directorate positions are occupied by women and only 7% are in executive directorship positions. In 2017, only one company listed on the Johannesburg Stock Exchange (JSE) had a woman CEO (Statistics South Africa, 2017). Furthermore, a mere 7.1% of these women held positions in the directory or presidency of institutions (Mathur-Helm, 2005).

While it is clear that the underrepresentation of female leaders pervades almost every work sector (Burton, 2019; Herbst, 2020; Liu et al., 2022; Pillay-Naidoo and Nel, 2022). Kaiser and Spalding (2015) state that underrepresentation is even more rife in male-dominated occupational environments. Male-dominated professions are defined as the professional work environment whereby traditional gender hierarchies prevail in the context of organizational policies and practices (Martin and Barnard, 2013). These environments hold the vision to maintain the marginalization of women within the working domain (Martin and Barnard, 2013). Due to male domination within these professional environments, they are structured in a manner that can be regarded as unsupportive toward women career paths and in essence create a male-dominated organizational culture (Martin and Barnard, 2013). Male-dominated professions include Science, Technology, Mathematics, Engineering, Financial and Banking sectors, which indicate a clear discrepancy in gender composition at the top hierarchical levels (Kahn and Louw, 2011).

Globally, a report presented by Price Waterhouse Cooper (PWC) in 2021 refers to the World Economic Forum Statistics and indicates that women are underrepresented when compared to their male counterparts in Mathematics and Statistics (4:5), Information and Communication Technology and Technology (2:5), and Engineering, Manufacturing and Construction (3:10), resulting in a significantly smaller pool of women in Statistics, Technology, Engineering and Mathematics (STEM) talent. In the South African context female leaders are significantly under-represented in male-dominated fields where only 11.9% of craft and related trade management positions are occupied by women (Statistics South Africa, 2017). Kaiser and Spalding (2015) further state that the main causes of underrepresentation in male-dominated professions can be explained by factors such as career breaks due to maternity leave, sexual harassment, male-leader bias, gendered structures within organizations, the imposter syndrome and extreme competition between women.

Hideg and Shen (2019) argue that the gross underrepresentation of women in leadership positions poses problems from both the business and ethical standpoint. From a business standpoint, failure to ensure equal women representation in leadership roles results in a missed opportunity to capitalize on the human capital of a large percentage of the workforce. Several authors (Hoobler et al., 2018; Hideg and Shen, 2019) advocate for the value that female representation adds for the bottom line of the organization. Hideg and Shen (2019) argue that from an ethical standpoint, lack of female representation continues to exacerbate gender inequality and serves as an obstacle for transformation of the workforce. Given the implications of lack of female leadership representation, authors (Webber and Giuffre, 2019; Pillay et al., 2022) argue that there is an urgent need for studies that investigate the factors that hinder and promote female representation in the workforce. While several studies have attempted to understand the barriers that contribute to female underrepresentation (Kaiser and Spalding, 2015; Hideg and Shen, 2019; Herbst, 2020) little is known about female leader’s experiences of career motivating strategies particularly in male-dominated fields. Webber and Giuffre (2019), argue that sociologists are also yet to explore the work conditions and experiences that help facilitate career growth for women. Particularly, the authors call for studies that focus on the role that supportive female work relationships may play in motivating women. This study answers this call by exploring the experiences of female leaders with regard to female workplace solidarity.

Solidarity can be defined as the ostensible ability to recognize and respect differences, such as race, class and sexual orientation, while expressing support for others (Littler and Rottenberg, 2020). Mavin (2008) states that there is a lack of research focusing on solidarity behavior and instead considerable attention has been directed toward the stereotypes that are believed to create division between women at work. According to Onojobi (2015) female solidarity is critical in combatting male hegemony which acts as a barrier to the unity between and among women. Furthermore, female solidarity allows for the entrenchment of genuine affection among women enabling them to stand firm within the workforce and combat division among the female workforce (Whitehead, 1984). Women experiencing gender inequality require individual as well as communal resources to foster their talent and the collective support from other women in the workplace can assist in this regard (Johnson and Mathur-Helm, 2011). Litwin (2009) supports the argument of centralizing female solidarity by explaining that female workplace solidarity creates opportunities for endorsement, mentorship and encouragement through women supporting the career progression of other women in the workplace. The authors argue that to truly eliminate the occurrence of gender bias within male-dominated fields, change in the context of supportive interaction between women within a workforce dominated by males is essential.

It is against this background that a need was identified to understand female leaders’ experiences with regard to the barriers to female solidarity behavior, the perceived benefits of female solidarity and the workplace interventions that can be implemented to increase solidarity among female workers within the context of male-dominated professions.

## A brief literature review

### Social identity theory and female solidarity

Review of literature of solidarity among women indicates that solidarity and unity between women in the workplace is a significant step toward eradicating gender inequality in the workplace (Webber and Giuffre, 2019). The rationale behind this is that when women stand together and show support for each other, they are in a better position to challenge the status quo and bring to light systemic inequalities in the workplace. In a review of research that looks at work environments that support women’s career growth, Webber and Giuffre (2019) argue that sociologists are yet to explore the work conditions that contribute to supportive female work relationships but instead there has been a fixation on exploring the stereotypes that are believed to create division between women at work. According to the authors there is a gap in research pertaining to the organizational environments and factors under which women support each other in the workplace and this warrants further attention. Adding to this, Derks et al. (2016) advise that future research should be conducted to develop interventions that assist in combatting self-group distancing, allowing women of socially devalued groups to easily combine their work and group identities. One such theory that explains how women may foster greater solidarity through intergroup identity is the Social Identity theory.

The concept of social identity was first conceptualized by Tajfel (1984) and emphasized the distinction between one’s personal identity and the identity experienced within group social situations with the assumption that the social identity is primarily derived from group membership (Brown, 2000). The Social Identity Theory is a concept that defines an interrelated group of psychological and social theories which focuses on how individuals identify with, or behave as, part of their particular social groups by adopting shared attitudes and behaviors (Tajfel, 1984). The theory is concerned with both the sociological and psychological aspects of an individual in relation to group behaviors (Tajfel, 1984). A review of literature on the role of social identify theory in explaining group solidarity indicates that social identity plays a significant role in determining intergroup relations. Everett et al. (2015) state that with reference to the social identity theory, a social group is a collection of individuals who believe that they belong to the same social category and by extension share similarity with each other. The stronger the perception of similarity the greater the social identity. The authors argue that strong social identification strengthens intergroup relations.

Adding support for the relevance of this theory to the current study, Baykal et al. (2020) argue that women who have a strong sense of social identity with other women in the workplace, experience feelings of belonging which occurs through assimilating the specific characteristics of fellow women. In the context of male-dominated work environments, the women leader who has a strong identification with other women in the workplace, is more likely to develop strong bonds with these women which in turn increases solidarity and supportive behavior. Likewise, the woman leader who lacks a sense of social identity may tend to display weak or no bonds with other women in the workplace and therefore has no identification leading to behaviors which are unsupportive or oppose solidarity with other women (Baykal et al., 2020). The reasoning behind this is that when women within male-dominated professions experience a high level of social identification, harmful behaviors like lack of support and unity toward other women, bullying and unhealthy competition in the workplace are minimized, ultimately allowing women to progress and advance within male-dominated professions. Using the social identity theory as the framework to establish the current argument, this study makes a valuable theoretical contribution by understanding the processes through which strong social identity fosters solidarity among women leaders in the workplace. In addition the study aims to offer a theoretical contribution by understanding the greater systemic and internal barriers that hinders solidarity among women in the workplace and sheds light on the ways in which female solidarity can benefit both the individual and the organization.

### Barriers to female solidarity

Empirical evidence confirms the notion that divisions among women in the workforce are prompted by negative career experiences of women in male-dominated work environments (Martin and Barnard, 2013). For instance, correlational data confirms that there is a known relationship between experiences of gender discrimination and women who distance themselves from other female leaders (Faniko et al., 2016). Female stereotypes such as the Queen Bee Syndrome reduce the likelihood of women progressing within the work environment in comparison to their male counterparts and are considered a barrier to female solidarity (Ellemers et al., 2004; Johnson and Mathur-Helm, 2011). Arvate et al. (2018) argue that the phenomenon is present among women who have low gender identification in their female reference groups due to their experience of underrepresentation and gender bias within male-dominated professions.

Additional research indicates the lack of recognition of gender inequality as a barrier to women’s solidarity in male-dominated work environments (Webber and Giuffre, 2019). The inability to recognize gender inequality in the workplace stems directly from aspects, such as neoliberalism within the workplace, a blatant disregard of women’s ability to succeed in a male-dominated environment and gender biases (Ringblom and Johansson, 2020). To address these trends identified within previous literature, the first area of interest for this study was to explore female leaders’ perceptions of barriers to female solidarity within male-dominated professions. Based on the discussion presented above, the following research objective has been proposed:

#### Research objective one

To understand female leaders’ perceptions of the workplace barriers that hinder female solidarity among women within male-dominated professions.

### Benefits of female solidarity

Female solidarity allows the entrenchment of genuine affection among women enabling them to stand firm within the workforce and combat division among the female workforce (Onojobi, 2015). Women experiencing gender inequality require individual as well as communal resources to foster their talent (Johnson and Mathur-Helm, 2011). This can be achieved by implementing female solidarity within the domain of male-dominated work environments. By employing these resources along with an attempt to increase female wellbeing within the workforce, solidarity will aid as a mechanism for women to enable the liberation of female employees (Onojobi, 2015).

#### Research objective two

To understand the benefits of female solidarity in male-dominated professions.

### Interventions to increase female solidarity

Author’s Webber and Giuffre (2019) identify possible resources/strategies to assist the way female relationships are formed within the work environment to ultimately increase female solidarity. These resources include: Transforming management strategies to increase gender stereotype awareness within organizations; creating platforms where women are celebrated to speak about their experiences within the workplace; and promoting participation in women’s affinity or networking groups (Seron et al., 2016).

Researchers suggest that by increasing female representation, the overall financial position of an organization will increase due to there being a direct correlation between women’s representation in leadership and various measures of firm performance (Hoobler et al., 2018). Furthermore, female solidarity is related to increased support, which can be used as a resource for women in male-dominated professions.

To address these gender inequalities and enhance female solidarity in a workplace context, the literature suggests various interventions to be implemented in assisting and encouraging women to better network and collaborate. In order to encourage women to share their experience of inequality within the workplace, Dobbin et al. (2014) suggest that diversity training should be made voluntarily for women. Diversity training can be implemented together with a formal mentoring program to provide women the opportunity to discuss occurrences of bias and the consequences and concerns associated with these occurrences (McGuire and Reger, 2003). Furthermore, research also suggests implementing mentoring and networking opportunities within the workplace to benefit the career advancement of women within the workforce (McGuire and Reger, 2003). However, it is believed that implementing such interventions, promoting support, encouragement and empowerment among women must be established within the structure and culture of the organization to mark a change in female representation within senior managerial levels (Williams et al., 2014).

#### Research objective three

To explore the various workplace interventions that can be implemented to increase solidarity among women in the workplace.

## Methodology

For the purpose of gathering expressive information, the researchers made use of a qualitative phenomenological approach to explore the perceptions of female leaders through the exploration of individual participant experiences. The implementation of the qualitative phenomenological approach allowed the researchers to explore the reality and world of the respondent (Creswell, 2009). Semi-structured interviews were conducted with 13 female leaders to gain an understanding of the perceptions of female leaders in male-dominated professions.

### Research approach and philosophy

Interpretivistic research can be identified as a set of unified assumptions about the social world or experience, providing a conceptual framework for the organized study of that world or experience (Zeller, 1995). Saunders et al. (2016) furthermore explain interpretivism by defining the paradigm as a position of philosophical orientation by understanding the way in which humans make sense and view their realities as well as the world around them. The paradigm is heavily dependent on the manner in which the participants of a study create their reality and their perception of their created reality (Creswell, 2009).

This paradigm assisted the researchers to gain an understanding of the perception and experiences of women in leadership positions regarding the barriers that women have to face to achieve solidarity. Furthermore, the paradigm also assisted the researchers in understanding the interventions that can be implemented to enhance female solidarity in Male-dominated professions. The paradigm allowed the researchers to understand the phenomenon of female solidarity in a natural setting.

### Data collection

Before collecting the data, ethical clearance was obtained from the Department of Human Resource Management at. The researchers relied on her own networks as well as the exploration of how participants were obtained in previous research studies to identify and approach the correct participants for this particular study (Wagner et al., 2012). Professional and social networks, such as LinkedIn, Facebook etc. were used to attract to obtain relevant participants based on their professions as well as positions to ensure the correct selection of a relevant sample. Platforms such as LinkedIn and Facebook were used to advertise the proposed study of the researchers with the purpose of obtaining participants.

Semi-structured interviews were conducted to gain an understanding of the perceptions of female leaders in male-dominated professions. To assist the researchers in the flow and structure of the interview, the researchers developed an interview guide which included questions about the barriers, benefits and interventions for female solidarity in male-dominated professions. The questions chosen to be included in the interview schedule were directly related to the research objectives and were included with the aim to, on reflection, address the objectives stipulated by the researchers. Questions, such as “How would you define female solidarity” and “What are the possible interventions to assist female solidarity in the workplace” were included in the study as they were expected to directly or indirectly assist in gaining insights into the females’ experiences within their working environment with regards to female solidarity. Furthermore, the researcher attempted to conduct a pilot interview before commencing with the data collection, which allowed the researcher to resolve any difficulties concerning the structure.

Within the context of the conducted study, the interviewer used a virtual platform, namely Microsoft Teams to conduct online interviews with participants. The platform furthermore allowed the researchers to capture the interviews in a recorded format. Transcribing the recorded interviews enabled the interviewer to analyze the data. The content analysis enabled analyzing large amounts of unspoken textual information to systematically identify or detect significant properties of its content through structured categorization (Creswell, 2013). Interviews were conducted until data reached saturation.

### Sampling

Non-probability sampling, according to Corbin and Strauss (2008), can be defined as the method whereby not all members of a population will have equal chances of participating in the particular study at hand.

Both purposive and snowball sampling were used. Purposive sampling is known as the method of non-probability sampling whereby the researcher strategically chooses the relevant participants following the study objectives (Corbin and Strauss, 2008). Given that the study was specifically aimed at understanding the experiences of women leaders with regard to female solidarity in the workplace, a sampling strategy that allowed the researchers to purposefully select participants was justified. In addition to purposive sample, the snowball sampling technique whereby the researchers used existing participants to recruit participants who met the sampling criteria through their personal networks was also implemented by the researcher (Miles and Huberman, 1994). The sampling criteria chosen within this specific research study included women in leadership positions in male-dominated professions, including occupational professions relating to Science, Technology, Engineering, Mathematics, Mining and Finance. Additionally, a *leadership position* is defined as occupying the position of supervision and upwards. This is due to the roles and responsibilities of supervisors often including mentoring, coaching, decision making and facilitating supervisors regarding their subordinates (Gray, 2018).

Lastly, participants needed to have at least 14 months experience in the leadership role due to having undergone a career journey to understand the value female solidarity will provide in increasing female representation in male-dominated professions. The researchers relied on their own network experience as well as the exploration of how participants were obtained in previous research studies to identify and approach the correct participants for this particular study (Wagner et al., 2012). Table 1 provides an indication of the demographic breakdown of the sample.

**Table 1:** Participant demographics.

Participant	Date of interview	Length of interview	Age of participant	Job title
1	3 February 2022	45 min	52	Portfolio Manager
2	4 February 2022	37 min	36	Chief Actuarial Officer
3	14 February 2022	45 min	47	Head: Wealth Service
4	16 February 2022	38 min	38	Actuary
5	23 February 2022	36 min	34	Chemical Engineering Team Lead
6	21 February 2022	28 min	45	Chief Actuary
7	07 February 2022	37 min	42	Chief Information Officer
8	2 February 2022	30 min	29	Team Lead
9	07 February 2022	38 min	32	Chemical Engineer
10	15 February 2022	35 min	37	Chief Information Officer
11	1 February 2022	39 min	52	General Manager
12	17 February 2022	56 min	49	Chief Information Officer
13	22 February 2022	35 min	43	Chief Data Officer

### Data analysis

After completing the data collection process, the researchers were required to analyze the data to make sense of the participants’ responses (Creswell, 2013). Clarke and Braun (2013) framework of thematic analysis was selected as the data analysis technique as it allowed for the researchers’ to recognize, analyze and report themes within the data collected. A step by step account of data analysis is provided below:

***Step 1-*** Familiarization by acquainting oneself with the content of the data through repetition:

In this step the researchers attempted to become familiar with the data using the transcription process. Written transcripts were digitally downloaded in the format of Microsoft Word to allow for corrections to be made. The data was re-read while listening to the recorded interviews whereby spelling and grammatical clarifications were made to the transcripts before the commencement of coding. The purpose of coding was to identify the initial codes and patterns in the data (Clarke and Braun, 2013). In the context of this particular study, the researchers familiarized themselves with the interview data collected from female leaders within male-dominated occupations by reading extensively through the interview transcriptions.

***Step 2-*** Coding the data by creating semantic and conceptual labels for critical aspects of the data that will ultimately allow answering the proposed research question:

Upon thorough inspection and familiarization of the data, a six-level approach to coding was followed by the researchers (Clarke and Braun, 2013). The general coding process commenced with initial familiarization of the data. The researchers generated a preliminary list of emerging patterns. The researchers thereafter commenced by initiating codes to the identified patterns in accordance with the presented research objectives. Boyatzis (1998) explains that the codes derived from the data would be the elements of data that are regarded as being of interest to the researchers in order to address the proposed research questions. Coding allowed the researchers to categorize her data into important groups, but not yet themes. The data collected on female leaders’ perceptions regarding female solidarity in male-dominated professions was coded by analyzing the critical aspects that arose from within Phase 1 of familiarization of the data.

***Step 3-*** Exploring meaningful and coherent patterns within the data by searching and identifying relevant themes:

During the third phase of the process, the researchers assigned themes to the codes through the process of combining related codes that were generated in Phase 2. This process allowed the researchers to formalize themes as well as sub-themes. The sub-themes arise when the main themes are classified into sub-categories, allowing broader themes to be split (Struwig and Stead, 2001). Due to the subject of the interviews specifically relating to female solidarity and the attempt to answer the proposed research questions, the researchers was intent on relating the themes to barriers, benefits and interventions related to female solidarity in male-dominated professions.

***Step 4-*** Determining the relevance of the identified themes concerning the full dataset and codes through revision of themes and defining the interrelationships between the identified themes:

The refinement of the themes identified in Phase 3 comprised this phase. The researchers did so by splitting, redefining and disregarding some of the themes, in order to ensure the relevance of the themes to the research objectives and questions. The relevance of the themes was measured against the research questions proposed by the researchers, related to the concept of female solidarity, specifically within male-dominated workplace environments.

***Step 5-*** Writing a detailed analysis of each identified theme by creating an informative label for the identified themes:

The researchers were required to examine all the responses captured in line with the identified themes in order to capture the essence of the data collected (Clarke and Braun, 2013). This added a level of patterned response as well as meaning to the data by naming and defining the themes presented in the data.

***Step 6-*** Integrating the extractions through analytical writings to present a coherent and contextual narrative of the qualitative dataset (Clarke and Braun, 2013):

The final phase in the thematic analysis process required the researchers to provide a final analysis and report regarding the narrative of the data in a manner that provides the reader with both merits as well as validity (Clarke and Braun, 2013). The report aimed at providing both a logical as well as a descriptive overview regarding the narrative retrieved from the data both within and across the derived themes (Clarke and Braun, 2013). It is of critical importance to ensure that the integrations and extractions gathered within the thematic analysis process related to the theme and concept of female solidarity, the barriers thereof, the benefits it may hold and the interventions to increase female solidarity to ensure the research objectives, were achieved.

### Quality assurance

#### Trustworthiness and reflexivity

Lincoln and Guba (1985) describe trustworthiness as the process in which the researcher ensures that the reality of the researcher is accurately captured, the data is contextually relevant and deemed as stable over time and circumstances, and the degree to which the data gathered can be reiterated by others as well as the degree to which the data gathered can be confirmed and validated by others. For the purpose of this study the researchers used the technique of field notes to take note of the non-verbal communication that the participant showcased during the interview, which may have contributed to the overall richness of the data gathering process. Saunders et al. (2016) further explain that an interviewer should identify possible biases within their own personal, political, social as well as philosophical beliefs that will possibly impact the method of collection and interpretation within their research process by using self-reflection.

In addition, the researchers used an audit trail described as the systematic process whereby documentation is maintained throughout all courses of action during the research process (Azungah, 2018). The trustworthiness and reflexivity of the study were enhanced through an audit trail indicating how all decisions were made. This allowed the researchers to be able to substantiate all findings and results obtained during the course of the research process (Azungah, 2018). The process reflected the communication between the participants and the researchers and also showcased all discoveries in the manner in which it was discovered (Schurink, 2003). Establishing the perceptions of the researchers that are related to the study can be identified and continuously checked to ensure personal perceptions were not being integrated into the data analysis process (Schurink, 2003). In addition to the communicational evidence, the researchers also included detailed descriptions of the sample used during the research process to ensure an accurate and transparent representation of the sample group (Azungah, 2018).

#### Transferability

The extent to which the finding of one study can be transferred and applied to other situations is defined as the concept of transferability (Merriam, 1998). Transferability in the current study is ensured as the findings are presented in an in-depth and descriptive manner regarding the context of the participant being described in order to allow the reader to judge whether the transferability of the findings presented is achievable (Miles and Huberman, 1994).

#### Confirmability

The confirmability of a research study is the extent to which the research can be confirmed and supported by others (Kane and Trochim, 2009). In order to gain confirmability within this research study, the researchers used the guidelines presented by Miles and Huberman (1994). These included providing detailed descriptions regarding the research process followed and in-depth explanations for the derivation of findings and results presented in the study, as well as addressing the effect and role of the researchers within the process, and addressing possible biases and assumptions (Miles and Huberman, 1994). Lastly, copies of the interview transcriptions were given to the participants to confirm that everything was transcribed correctly and nothing was misconstrued (Kane and Trochim, 2009).

### Research ethics and authorization

The main objective of the researchers were to refrain from any harmful activity in the process of conducting a research study (Brinkmann et al., 2018). It is therefore of critical importance to implement ethical considerations during the course of the research (Brinkmann et al., 2018). The researchers obtained ethical from the Ethics committee at the Department of Human Resource Management, University of Pretoria (MMD/2021/26). Due to the use of purposive and snowball sampling, the researchers’ did not approach a specific organization but instead posted a participant recruitment advert of professional networking sites for potential participants to respond to the advert if interested. Participants who met the criteria were informed of the time and logistics surrounding the interview and sent a link to join the session. The researchers ensured that confidentiality was maintained by providing the interviewees with a consent form to sign prior to the commencement of the data gathering process. The consent consisted of an agreement by the participant to take part in the study as well as permission to use the data presented by the participant in the process (Leedy and Ormrod, 2010). The data gathered during the research study was solely used for the intended purpose of the research study and will furthermore be secured for a period of 5 years after completion of the research study (Babbie and Mouton, 2001). In addition, the researchers adhered to ethical guidelines as proposed in the University of Pretoria’s code of ethics. The disclosure of the research intent is of critical importance to ensure ethicality is maintained. Permission to record and use the information gathered from the interviews was gathered in both a verbal as well as written format (Punch, 1986).

Furthermore, in adherence to the right of privacy and confidentiality of the participants, the researcher ensured that the personal details of each participant remained undisclosed, and the researchers ensured anonymity as well as confidentiality throughout all stages of the research process (Bulmer, 1982).

Finally, the participants were informed that participation during the research study will be completely voluntary and should the need arise to withdraw from the process, they have the right to do so (Saunders et al., 2016). All participants were informed that any withdrawal during the process would not lead to negative implications, and should the need arise, assistance would be provided in the case of any harm suffered during the process. A debriefing session with participants after the interview allowed the researcher to clarify any misconceptions as well as questions that may have arisen during the course of the study.

## Results

Table 2 indicates the key themes identified for each research question.

**Table 2:** Systematic themes diagram.

Research objectives	Understanding female leaders’ perceptions of the workplace barriers that prevent solidarity among women in male-dominated professions	Understanding the benefits of female solidarity in male-dominated professions	Exploring the various workplace interventions that can be implemented to increase solidarity among females in the workplace
Research questions	What are women leaders’ perceptions of workplace barriers that prevent solidarity among women in male- dominated professions?	What are the benefits of female solidarity in male-dominated professions?	What workplace interventions can be implemented to increase solidarity among females in the workplace?
Main themes	Perception of workplace barriers that prevent female solidarity	Benefits that female solidarity hold	Workplace interventions to be implemented to increase female solidarity among women in the workplace
Sub themes	Unfair workplace behaviors Generational beliefs Societal expectations Organizational culture Old boys club Stereotypes and Stigma’s	Career shaping mentorship Female recognition Female representation Female support	Networking Transforming company culture Socialization Mentorship

Following the data collection process whereby the researchers used semi-structured interviews, the data was analyzed to identify themes relevant to the above-mentioned objectives of this study. The researchers included quotations extracted from the participants to substantiate any inferences that were drawn. For reporting purposes, each verbatim quote captured from participants is coded to indicate the participant numbers, e.g., P1 = Participant 1, P2 = Participant 2 and so forth. The themes identified during the data analysis phase are presented in accordance with the research objectives listed above.

### Themes emerging from research objective one: perceptions of workplace barriers that prevent female solidarity

Within this study, the participants identified the predominant barriers within male-dominated workplaces as unfair workplace behaviors, generational beliefs, societal expectations, organizational cultures, and stereotypes and stigmas.

#### Unfair workplace behaviors

Unfair workplace behaviors refer to the participants’ perceptions with regard to the less favorable treatment of female workers as opposed to male workers in the same circumstances. Many participants provided examples of workplace behaviors that could be deemed as unfair and in essence prevent females from achieving solidarity:

*That was when I was frustrated and angry in a boardroom set-up because I am very orderly. Being an accountant by training. And then arguing with the sales director. Because I was only one of 3 women on the board and the only female to stand up against our male counterparts, I was treated differently.* (P7).

*And then. I got worked up because he was really, he was wrong in what he was presenting and he turned around and said, what are you menopausal? Exactly, and I was just like gobsmacked. I did not gain any support from my female colleagues, except for one. How can we expect to progress in this environment if we cannot stand up for one another?* (P6).

The unfair behavior mentioned created division among the female workforce because the behavior mentioned was only experienced by certain women within the workplace. Some did not believe that the unfair behavior had happened. It can be seen that the inability of women workers to support other women employees when they are voicing their disagreement with unfair workplace behaviors creates a divide between women in the workplace.

#### Generational differences

This subtheme can be defined as the differences in values, beliefs and opinions between individuals from different generations of women within the working domain. Participants delved into the mentality of the older women generation with regards to workplace behaviors and the contributions women were seen to make; this, in hindsight, created division among women in the workplace due to the perceptions they carry based on their generational beliefs.

*Maybe it's perception, but that people feel, and I think a lot of that comes from an old mentality in terms of the way the workplace used to be defined. And they didn't see them (women) as contributing too much to the workplace, so I think that stigma is very prevalent amongst your older management. I've encountered that many times.* (P3).

*Then they started working and you know, women were generally seen as sort of the “stay at home look after the kids” kind of people.* (P4).

Within the extractions based on generational differences, it is evident that there are unshared perceptions among the older and younger women, creating disagreement among older and younger workers that, in turn, decreases solidarity.

#### Societal expectations

Societal expectations refer to the general standardized behaviors that women are expected to uphold in work and family domains. The first expectation identified was that of women being expected to take on the role of caretaker within their households, while trying to maintain success within their career domain.

*Understanding the balance you are trying to bring in. You can't have the conversations of “I feel guilty as a mother who's working” because of the hours I'm spending at work and how I'm compromising my child. A male doesn't understand that. Uh, some female knows that. A female potentially has had that guilt trip herself and has worked through that guilt trip but it is not always the case* (P1).

*Uh, so as an example, I think that our little family if someone looked from the outside in. It's not a traditional type of family setup, but thankfully there are more of those setups coming in, which makes it easier. But as an example, like I never cook, he's he always cooks, 'cause he's done earlier so he can help. However, some women are completely against this setup and will judge you for it* (P10).

*You step out of that voting cubical and you go into society and work. All of the discrimination still exists. Society still expects women to carry certain responsibilities.* (P1).

#### Organizational cultures

Organizational cultures are defined as collective basic behaviors and assumptions that employees share to, over time, successfully adhere to internal and external organizationally relevant problems. Three participants indicated that the organizational culture within an organization can be a detrimental barrier to women working together and experiencing solitude within their work environment.

*Create a new team that aligned with my cultural objectives. My uh, my vision of what this area should be like. And if you think of it, I am one person in a workforce of 12 000, how am I meant to create this team of women working together?* (P1).

Two participants indicated that conflict within the work environment between women can create an unfavorable organizational culture and ultimately create division among women in achieving the goal of solidarity.

*When there is competition between women, it just results in absolute the opposite, whatever the opposite definition is of solidarity, it results in that because as soon as women start feeling that they need to compete with one another and there's only one place for one of them at the higher level, then it just every sense of solidarity collapses…… And then when you start getting that feeling within your workplace, you start competing against other people and you start withholding your contribution because you feel that you don't want to help someone else. After all, they might get promoted.* (P13).

#### Stereotypes and stigmas

Stereotypes and stigmas are defined as the creation of assumptions regarding a certain group of individuals due to broad statements or ideas of the collective group they belong to.

*I don't want to say there's no space but then we become so guarded about our emotions. Oh my goodness you can't cry….Age is a big deterrent. You know that’s how some might feel. That's dumb because there's a female on the other side of the screen that's in her late 20s or early 30s. I don't need to say they won’t even listen to you, you're a child.* (P2).

*And then of course it's the prejudices of, depending which industry you end, the sort of make-up of the management and their attitude towards improving workplace equality…Women themselves go into positions and think that they shouldn't even try because there is going to be such pushback due to the fact that it's a predominantly male environment in a sort of predefined male job. As for women, historically they didn't really see them as contributing too much to the workplace, so I think that stigma is very prevalent amongst your older management. I've encountered that many times.* (P6).

Furthermore, participants mentioned the manner in which certain behaviors are received when it is enacted by female leaders as opposed to male leaders. Such discrepancies may also cause barriers for women when trying to achieve female solidarity in male-dominated professions. Some women think that when showcasing these behaviors (generally displayed by males) women are attempting to “act like men” rather than embracing their female characteristics. These conflicting opinions lead to conflicting views among the female workforce, leading to a decrease in female solidarity in the workplace.

*If you disagree in a male-dominated sort of environment, you have to be very careful about how you do that. And often you know we are, let's say if X person had said the same thing I had but with a male voice I think it would have been received differently, so it's tremendously difficult because you're already feeling on the back foot and then you almost can get punished because you've seen as being too aggressive. And I think that summarizes the situation where I think assertiveness is the word I was looking forward to. That's easily often interpreted as aggression for women. I see that time and time again.* (P13).

*You know, there's a far higher probability that it's true. Right. Uh, but I think as women we don't handle the competition with other women. Wow. It's also really interesting that if two women disagree, it's considered bitchiness. If two males disagree with each other in a meeting, it's what a healthy discussion they are having*. (P1).

### Themes emerging from research objective two: benefits of female solidarity

The benefits that female solidarity hold can be defined as the advantages gained that assist all genders within male-dominated professions to improve the overall work environment. Within this study, the participants identified the benefits of female solidarity within male-dominated workplaces as career shaping mentorship, female recognition, female representation as well as female support.

#### Career-shaping mentorship

Career-shaping mentorship can be defined as a tool for not only females, but also for males to create guidance amongst the workforce to turn their visions into realistic and tangible goals.

*I think that's why I mean. The reason they are successful is that those mentoring relationships are very authentic. So when you do go and do something on the weekend, you do want that individual to come with you and shape their reality as well. You want her to progress and see the benefit of being that role model to her* (P10).

This participant also indicated the benefits of female solidarity in her career.

*I honestly think this female person shaped the rest of my career, she was the kind of person that cared very deeply about you being successful. She went out of her way to make sure that you will be able to have the tools to progress* (P10).

#### Female recognition

The theme known as female recognition can be defined as the ability to acknowledge women’s perceptions as well as praise for their work within Male-dominated professions.

*I think that speaks a lot to why there's that sort of comfort for the sort of woman in South Africa, you know that we are recognized and that there is space made for women to participate in those sort of managerial roles.* (P2).

*And I know very, very cool, and I mean they outshine the males like in everything. They're always being awarded, you know, from their industry and stuff like that. So that's fantastic to see. It's very inspirational for the rest of us.* (P3).

#### Female representation

Female representation in the context of this study can be defined as the ability to acknowledge the presence of a female workforce within the workplace and affording women opportunities for representation.

*I think as we see you know, women are being accepted into different roles. And I think that that's gonna encourage our youth to try and to...To push those boundaries, there are plenty of women that those two female rowers have been the first female to ever cross the whole Mediterranean, I mean or the Atlantic. Sorry for the fact that they are inspiring young women who are in a very male-dominated type of sport. This is now, it's the news and they're inspiring young women to also just break free from stereotypes* (P3).

#### Female support

Female support for this study is defined as the process in which females assist one another in elaborating and recreating their abilities by accomplishments in the workplace what was previously denied to them. All the participants agreed that the most significant benefit of female solidarity in the workplace is female support in a male-dominated environment.

*Uh, we don't speak about the next woman's crooked crown. Walk up to her and straighten her crown* (P13).

*Lucky we are so blessed to be in an environment that uplifts each other, whether it is the males uplifting the women, whether it's the women uplifting the women.* (P4).

*Put on corporate pants and show up knowing that hey there is somebody that's got my back there is somebody that's just holding.* (P7).

The majority of the participants indicated that the emotional support women receive is a substantial benefit increasing female solidarity in a male-dominated environment.

*It reminds you that you're not crazy. That is it.* (P4).

*And all of that, and I think sometimes just you know, ears or shoulders to cry on. If I can say that you know, it can be very difficult, especially those. And you know, you get this perception that's.* (P11).

You know, and when I speak emotional support because somebody is in their mid-30s and that has two little kids and that cannot work 12 hours a day.

*They can have that now done in an environment in a space where they feel. I'm not judged. Where they can feel like I can't talk about my feelings. I can talk about my personal situation, but also know that I can wipe my tears.* (P13).

*And then all you do is you go and talk to somebody who has done this for 500 years and is also a female perhaps. And as you know experience and a bit more calm and perspective to share with you.* (P6).

#### Networking

Networking for the purpose of this study is defined as the process of interacting with work colleagues by establishing and developing both professional and social relationships.

*I do think mentors are a good concept, but I think mentors should come from themselves, right? I think we need to create initiative with individuals where it's an open-ended thing. But there's always someone that knows more and that we actually end up interacting with that before must level up a bond and I think it's actually up to that individual to make that link*. (P6).

*I think I think networking is definitely important.* (P3).

*I think that that's it. It's a few initiatives. Also, at the current company that I work at, we have a women's group where all the women within the organization are invited to a 30-minute session.* (P5).

*It also comes from female colleagues, right? So if I think about the women that I work with, I have a great relationship with them.* (P9).

The implementation of female networking interventions will allow women to collaborate and build workplace relationships, in essence driving female solidarity among the female workforce in male-dominated professions.

#### Transforming company culture

This subtheme is demarcated as the movement of a current known state of an organizational culture toward a potentially unknown state.

*It’s a much larger social issue that we're dealing with, but. I feel that. In your own sphere of control, if you are in a position of leadership. You need to create the environment that prevents this 'cause that's your responsibility at the end of the day, 'cause you hold a leadership role. Uh and? You can only change what you can control. So you need to, you know, each one of us, I think has to stand up and fight for rights and whatever else it is and create these environments.* (P1).

*And it's also because basically when they come in, they sort of introduced to that culture. So I think it's more about changing the culture of the company so that it is more inclusive in terms of women.* (P6).

*That initial decision that I will not break down. Uhm, relationships between other women and I will be bold to say a supportive culture irrespective of gender.* (P7).

The last participant specified that a culture should be created within a male-dominated environment that is not competitive in nature.

*Little things are actually big things they do. You define your culture. I think that you have to have a culture that is not cut-throat.* (P13).

#### Socialization

The socialization intervention for the purpose of this study can be described two-fold. Firstly, interacting with other females in an informal manner. Secondly, the process of introducing an idealization of norms for behavior in society.

*And I think it's not just so it's almost taking the solidarity outside of the turnstiles.* (Participant 6).

*For me, this is a. To overcome this, I think we have to have. Honest conversations. You know you. You need to have an environment. That doesn't penalize you for having that conversation, so. If I am. In a meeting where my position. Is. More senior. Or my position? Is equal. To the people in that room. I can deal with the cutting me off. Right. Because I will just turn around and go, excuse me, don't be rude. I am still speaking.* (P1).

*We need to start. Being kind of sounds firstly and accepting we can't do everything that's expected of us and. We need to raise our sons. To be respectful, 'cause. And we need to educate them as to what it is we need to ensure that they understand what equality truly means. You know, at the end of the day.* (P12).

Furthermore, Participant 11 indicated that the intervention of socialization can be implemented in a manner of creating informal relationships with colleagues outside the work environment.

*Uhm, I don't know of any other examples that I can think of Yeah. I think one example is some people have like little exercise routines together, but that's maybe just the normal work phenomenon. Uh. And then? Yeah, like after work, just going for run 'cause. We live in like a small town, so it makes things.* (P11).

The last aspect of socialization was addressed as an intervention by participants as they indicated that, within the workplace, female solidarity should be communicated to increase an awareness of its importance.

*So I think there's a lot of things that we could do unless being honest and. Just in terms of like setting up more kind of structures where it's safe spaces I guess and more of a trusting relationship with other women where you know if you're struggling with something you can grow to someone. So I mean that could be more formalized through, like setting up events that encourage ladies to get together, and you could even have guest speakers like where there's somebody you know who's done well and kind of a good role model externally, or even you know locally.* (P7).

*I think probably, maybe subtle things. So maybe having weather in smaller groups. Branches or lunch is all I'm like, exercise, exercise group, like maybe like a running group, but they said on Wednesdays or Thursdays like all the women go walking like anyone that wants to go can go walking.* (P9).

*I heard very much those seeing teams come out in a conversation really like it. Informal conversation*. (P2).

*They are support groups and social groups, and I don't know what else. There's also a mentoring system at (company name).* (P10).

#### Mentorship

The final intervention subtheme can be defined as providing guidance and assistance by an experienced leader or employee in an organization. Mentorship is a key aspect of relationship building, therefore, in order to build relationships among women within male-dominated professions, this intervention will allow female solidarity to prosper.

*Yes, absolutely. So especially for the grads when they come in, they are allocated to a development team and they are definitely mentored. In fact, we've got a fantastic young female who joined us two years ago and she is just brilliant. So she came through the grad program. Only female out of all of them. She was allocated to our team and she's just gone from strength to strength and they recently employed her.* (P6).

*Globally that person could present from (company name) and I think, like, uh, mentorship type of a program can be quite nice where you kind of pair people app and you know, maybe a senior leader with you know someone more junior that's still working up through their studies and that and can help with the whole thing and uplifting each other. And I think just also being more aware.* (P7).

*If you have a political player that does that and is very successful, then it encourages others to do the same.* (P13).

## Discussion

Findings from the analysis suggests that unfair workplace behaviors are very often experienced by women, especially in male-dominated professions and can be seen as a significant barrier to solidarity in the workplace. In the literature, Dellinger and Williams (2002) discuss the differences in perceptions with regards to gender inequalities and the impact thereof on female achievements at work. The process through which unfair workplace behaviors leads to counterproductive solidarity behaviors occurs through the opposing perceptions among the female workforce with regards to unfair workplace behaviors. In most cases, women in the workplace has become accustomed to certain unfair workplace practices that they no longer see it as being unfair. According to Webber and Giuffre (2019), women are discouraged from engaging in ‘office politics’ and to overlook the gender inequalities they experience within the workplace, but are instead asked to focus on individual action and performance. Britton (2000) indicates that women habitually ignore or ultimately decide to dismiss unfair discriminatory behavior within the work context. This is done in an attempt to ignore the systemic barriers of gendered discrimination by rather focusing on their own performance to ensure career progression. The individualistic approach taken by women in this instance creates a divide between the female workforce as some women believe that women should stand in solidarity against this unfair behavior while others believe that the focus should be on individual performance and not changing what is considered beyond their control. This ultimately inhibits the progression of female solidarity in male-dominated professions. Hurst et al. (2018) note that organizational gender biases are rarely overtly faced by women, but are rather experienced in a covert manner referred to as second-generation gender biases. These biases are difficult to expose and are often argued to be a misconception by the opposing party. Entrenched in the denial of unfair workplace behaviors are the beliefs and assumptions that behaviors that are deemed discriminatory are simply the perceptions of women in male-dominated professions or that women are being overly sensitive to these behaviors (Webber and Giuffre, 2019).

In addition to unfair work behaviors, findings of the analysis identified generational differences a barrier to workplace solidarity for women. Seminal research on female solidarity by Whitehead (1984) states that female solidarity is constructed based on concrete interests shared among women. In addition, Whitehead (1984) continues to explain that divisions are created between women due to developmental lifecycles, female roles and the work transgressions among female employees. These divisions are created among women who embrace differing opinions regarding the phases they hold within their careers. Generational differences in the context of this study emerge when differences occur in values, beliefs and opinions of women from different generations within the working domain. These differences often lead to conflicting opinions in the workplace, creating an ultimate barrier to achieving shared interest among women in the workplace (Whitehead, 1984). When women’ roles within the workplace deviate from expected norms, gender differences may arise between those women who challenge the norms and those women who are highly sensitive to these norm violations (Sheppard and Aquino, 2016). Results within the study indicated that the traditional unspoken perceptions associated with certain genders (such as males being the breadwinners) create a divide among the workforce due to the younger generation having a different perception of gender roles in the workplace and society.

Closely related to generational differences in the barrier of societal expectations on how women should behave. Among these behaviors in the methods of communication which are perceived differently when showcased by women as opposed to similar behaviors exhibited by males, Mavin (2008) contends that women in leadership face difficulty when they “act like men.” They face the violations of expected gendered behavior, but if they accentuate feminine characteristics, they are faced with a devalued work status. Women with a more traditional mindset view these “masculine behaviors” such as being assertive or challenging the status quo as unacceptable as it goes against what is typically expected from women in the workplace. This difference is societal expectations creates divide between women employees.

The second objective of the current study was to explore the perceived benefits of female solidarity in the workplace. In essence, findings of this study and existing literature (Niler et al., 2020) suggests that female solidarity may hold beneficial outcomes for women in organizations, particularly focused on Science and Technology. Woolley et al. (2010) discuss the significance of interpersonal relationships between male and female team members and conclude that all-women teams exhibited increased egalitarian behaviors in comparison to their male counterparts, suggesting that female involvement in male-dominated professions enhances collaborative efforts as well as group performance (Woolley et al., 2010). Results within the current study confirm these suggestions and show women elaborating on their experiences of other female executives providing guidance and mentorship to anyone in the organization, engaging openly with women to provide their inputs. Career shaping mentorship is a benefit that can be seen as a tool to assist with building successful career relationships among female employees. These relationships can assist by encouraging women to turn their visions into realistic and tangible goals (Hurst et al., 2018). Lyngsie and Foss (2017) advocate that top managers influence the values, visions and goals of their subordinates within an organizational context. In instances where women support each other, specifically through solidarity, the mentorship relationship in essence creates and inspires support among lower-level employees (Lyngsie and Foss, 2017).

Female representation was also identified as a perceived benefit of workplace solidarity in the current study. By increasing the solidarity among female employees, specifically in male-dominated professions, women begin to support each other, they have a collective voice and are represented in the workplace. Results indicated the beneficial impact of multiple women in male-dominated professions for other women in the workforce by pushing the known boundaries and stereotypes associated with these environments. In a study conducted by Hyde et al. (2008) results suggest that the lack of participation of women in male-dominated professions was not due to intellectual abilities, but rather social influences on women to engage in science and technical fields. However, Niler et al. (2020) state that collaborative efforts and group performances increased in instances where women are involved in science teams. This suggests that female representation in work teams is an identified benefit of female collective efficacy that will in essence positively impact team performances (Niler et al., 2020).

Finally, the findings of the study related to workplace interventions to enhance solidarity found that found several interventions that can be used to promote solidarity in the workplace. Firstly, female networks which are viewed as the relationships that individuals will develop and use whether it be consciously or subconsciously to progress within their working enterprise can be used (Hampton et al., 2009). The results of this study indicated a relationship between interpersonal networking interventions and increased female solidarity in the workplace. Networking allows the opportunity for female-to-female interaction that also increases the chances of female solidarity. These relationships are often characterized by high levels of mutual commitment and trust by developing shared experiences (Cromie, 2000). Networks provide women the opportunity to share experiences and find common ground, an important aspect to build female solidarity, emphasizing the results obtained in the study to implement networking as an intervention to strengthen female solidarity within a workplace domain.

In addition to networking, a critical intervention identified to increase solidarity within the workplace is transforming or adjusting the company culture. The first step identified was creating a more inclusive culture within male-dominated working domains. Webber and Giuffre (2019) suggest that this can be done by implementing implicit bias training to identify the biases that will affect the exclusions of certain genders within the workplace. Correll (2017) suggests that there should be a shift from individual decision making toward organizational processes whereby the ‘culture of inquiry’ is promoted. The culture of inquiry allows for larger institutional culture change that will effectively increase female solidarity at work.

## Implications, recommendations for future research and limitations

With regards to the implications of the current study, organizations are encouraged to create awareness through relevant training–such as bias training. Organizations as a whole will also be required to consider their cultures and adopt workplace cultures that promote rather than ignore female solidarity and collaboration. Furthermore, the study provided practical interventions that can be implemented within organizations to assist with the attempt to promote female solidarity in male-dominated professions. The remaining interventions to be practically implemented within the workplace is networking and mentorship. A practical recommendation to introduce these interventions in the workplace would be to implement a companion system, similar to that of a ‘buddy system’ implemented in most organizations. This would allow women to have access to an individual in whom they can confide on a professional level. In addition to the above-mentioned recommendations, the importance of female solidarity should also be socialized–not only in the workplace, but in a societal sense. By creating awareness, the quest to improve female workplace relationships should increase and eliminate stereotypical perceptions.

As a recommendation for future research, scholars are encouraged to consider mixed method approach which may be beneficial to implement the interventions in a practical manner by corroborating both qualitative and quantitative findings. In addition to that, the development of a tool to assess authentic female work relationships as well as female solidarity in the workplace may be beneficial.

The following limitations were identified in the study:

The first limitation that the researchers experienced was the attempt to interview participants on the backend of the COVID 19-pandemic. This posed many challenges to the researchers, such as finding participants that were willing to conduct an interview. The only manner in which the researchers was able to complete the data collection was to propose virtual instead of face-to-face interviews to be conducted with the participants. For future recommendation the researchers proposes different methods of gathering insights such as focus groups. This will not only allow the researchers to gather insights from the participants, but will speak directly toward the cause of allowing women to gather in groups and share their experiences. This method will directly assist in an attempt to increase a feeling of solidarity among female workers and create networks among women of different spheres of life.

Another limitation experienced by the researchers was that of geographical location. Due to the social network the researchers used, she was limited to participants within the South African region. The study has tremendous potential to be expanded past the borders of the country of origin. Therefore, as a recommendation for future research, the researchers proposes expanding the study beyond the borders of South Africa to gather a fuller understanding of female perceptions in multiple locations globally.

In the process of data gathering, time constraints did not allow the researchers to conduct follow-up sessions with the participants in order to validate their initial responses to the proposed questions asked as well as their interpretations of the answers that were given. To respond to this limitation, the researchers suggests that researchers who want to use a similar approach, such as interpretivism, to incorporate time planning into their study to clarify the interpretations gathered from their participants.

## Conclusion

This study shines light on the importance of authentic working relationships among women through the concept of female solidarity, by exploring the perceptions of female leaders in the workplace about the building of positive workplace relationships with each other. These perceptions create an understanding of the benefits female solidarity hold not only for female career advancement but for the overall organizational success of male-dominated work environments. Furthermore, this study will contribute to the body of knowledge on the development of interventions to increase female solidarity within the workplace, enabling an increase in the representation of women within the domains that are regarded as male-dominated. This study catalyzes female leaders in the possible obstacles they may face in their quest to build female solidarity which serves as a motivating factor for women’s career progression. Furthermore, it will empower women with the necessary interventions to be implemented to increase their working relationships and create solitude among the female workforce.

## Data availability statement

The raw data supporting the conclusions of this article will be made available by the authors, without undue reservation.

## Ethics statement

The studies involving human participants were reviewed and approved by the Department of Human Resource Management, University of Pretoria. The patients/participants provided their written informed consent to participate in this study.

## Author contributions

This article was adapted from the Master’s thesis of CV, who executed the research, while DP-N was the study leader and provided conceptualization guidelines and editorial inputs. CV prepared the first draft of this article while DP-N made substantial revisions and editing. All authors contributed to the article and approved the submitted version.

## Funding

This study was executed through personal funds.

## Conflict of interest

The authors declare that the research was conducted in the absence of any commercial or financial relationships that could be construed as a potential conflict of interest.

## Publisher’s note

All claims expressed in this article are solely those of the authors and do not necessarily represent those of their affiliated organizations, or those of the publisher, the editors and the reviewers. Any product that may be evaluated in this article, or claim that may be made by its manufacturer, is not guaranteed or endorsed by the publisher.
